# Digital Replantation In Hours Versus Out of Hours: A Retrospective Review of Outcomes

**DOI:** 10.7759/cureus.73965

**Published:** 2024-11-19

**Authors:** James Holland, Ivy Kuo, Malin White, Kim Borsky, Francisco Moura, Remy Rees-Goddard, Caroline McGuiness

**Affiliations:** 1 Plastic Surgery, Salisbury District Hospital, Salisbury, GBR

**Keywords:** digital amputation, digital replant, hand trauma, ischaemia time, microvascular surgery, success rate, timing of surgery

## Abstract

Introduction: The British Society for Surgery of the Hand’s (BSSH) Getting It Right First Time (GIRFT) guidelines recommend that surgery for traumatic amputations of the digits is ideally performed during normal working hours even if this results in a delay of up to 24 hours.

Objective: To compare current practice against the BSSH GIRFT guideline regarding the timing of digital replantation and to compare the success rate of replantation performed within working hours (same or next day) and outside of working hours.

Materials and methods: A single-centre retrospective analysis of two years of digital replantation was performed. A comparison was made between success rate of those operated within and outside of normal working hours. Secondary outcomes included operating time, rate of revision surgery, rate of interposition vein grafting, and the effect of the mechanism of injury on success rate.

Results: A total of 32 digital replantation attempts were included in 21 patients. 71.9% of cases (n=23) were performed within normal working hours, demonstrating good compliance with GIRFT guidelines. The success rate of digital replantation attempts was 68.8% overall (n=22). The success rate of same day in hours replantation was 66.7% (n=6), 78.6% (n=11) for next day in hours replantation, and 55.6% (n=5) for digital replantation attempts made out of hours. Revision surgery was required in 28.13% of all replant attempts (n=9). 55.56% (n=5) of cases requiring revision were ultimately successful. The rate of vein graft use was highest in cases taking place same day in hours (75%; n=3) and lowest in cases same day out of hours (22% n=2). The average operating time per digit was 5.5 hours for same day in hours cases, 4.8 hours for next day in hours cases, and 7.1 hours for out of hours cases.

Conclusion: Digital replantation during normal working hours was associated with higher success rate although no statistically significant difference was observed between timing groups. Replantation during normal working hours was associated with shorter operating time per digit, and lower revision rate, although statistical testing was not performed.

## Introduction

Traumatic amputations of the digits present an interesting clinical and surgical challenge for plastic surgery departments worldwide. The global age-standardised incidence of thumb and non-thumb digital amputations is estimated to be 24 and 56 per 100,000 respectively [1]. The same study observed the highest rates of hand trauma in higher socio-demographic indexed nations and the highest rate of digit amputations was seen in North America and Europe. The British Society for Surgery of the Hand’s (BSSH) Get It Right First Time (GIRFT) guideline presents a national recommendation for the management of traumatic amputations of the digits in the United Kingdom (UK) [2]. This guidance recommends that traumatic amputations of digits be referred to a specialist plastic surgery department equipped with the facilities and expertise to attempt digital replantation if appropriate. It recommends that a replantation attempt should be made by a trained surgeon with extensive microvascular surgery expertise. They should also have access to an appropriately trained team of theatre staff. It suggests that digital replantation should be performed within normal working hours where possible, even if this necessitates a delay of up to 24 hours. 

The objectives of this study were threefold: (1) to assess the current practice of digital replantation at our centre and compare it to the BSSH GIRFT guidelines regarding the timing of replantation, (2) to compare the outcomes of digital replantation performed during normal working hours with those conducted out of hours, and (3) to share our experience in managing digital replantation cases at our regional hand trauma centre.

## Materials and methods

This retrospective observational study aimed to analyse outcomes of digital replantation from a single plastic surgery regional referral centre over a two-year period. A study period of two years was chosen in order to capture an appropriate number of recent cases of digital replantation to analyse their outcomes. A retrospective review was conducted of all cases of digital replantation attempted at Salisbury District Hospital Plastic Surgery department in the United Kingdom between May 2020 and October 2022.

Cases of digital replantation were identified by screening for relevant clinical codes on the trust's electronic patient medical record system and by colleague survey. Potential cases were screened by two independent assessors to ensure that only relevant cases had been identified and included. Operations were completed by different surgeons within the plastic surgery department during the study period. All cases were performed by a senior plastic surgeon. All patients who underwent digital replantation for traumatic amputation of the digit(s) during the study period were included. For cases where digital replantation was delayed, amputated digits were wrapped with moist gauze, and stored in a plastic bag within a medical fridge. Advice issued to referring hospitals when accepting transfer of care was to wrap amputated digit in moist gauze, place in a plastic bag, and submerge in a plastic bowl filled with ice and water. No patients undergoing a digital replantation for traumatic amputation of the digit(s) were excluded, provided they occurred within the study period. Patients were excluded if immediate terminalisation was performed before attempting a replantation, due to the extent of injury. In all of these cases, a senior surgeon had assessed the injury and deemed a replantation attempt to not be a technically viable option due to the extent of injury. Once all cases of digital replantation had been identified during the study period, a thorough review of medical notes and clinical imaging was undertaken. This included a review of paper notes from initial clerking through to final postoperative clinic follow-up, X-rays on local radiology software, clinical images, notes and letters on the hospital’s electronic patient record. All data was tabulated in Excel (Microsoft, Redmond, WA, USA) using a predefined extraction form. If data was incomplete or unavailable, a standardised approach was employed which included consultation with colleagues who were involved in the procedure and/or direct communication with patients via telephone. Two cases had incomplete medical records so the above protocol was followed to ensure comprehensive and complete capture of all relevant data.

In order to meet the specific objectives of this study, data collection methods and outcomes were predefined and agreed amongst authors. Basic demographic data was recorded for each patient to include hospital identification number, sex, and date of birth. The primary outcomes analysed in this study were the timing of surgery (classified as either in-hours or out-of-hours) and the success rate of digital replantation. Surgeries that began between 06:00 and 17:30 were categorised as surgeries within normal working hours. Surgeries starting outside of this window (between 17:30 and 06:00 UTC), were categorised as out of hours procedures. An outcome of successful replantation was defined as a digit that remained healthy and viable at four weeks postoperatively. This information was usually obtained from clinical correspondence generated from outpatient postoperative reviews. Secondary outcomes of digital replantation that were assessed included total operating time (per digit), the rate and success of revision surgeries following digital replantation, the rate of vein graft usage, and the association between mechanism of injury e.g. sharp or crush injury, and success rate of replantation.

Success rates of digital replantation attempts were compared between timing categories and statistical testing was performed using Fisher's exact test (due to the relatively small sample size within comparison groups). Descriptive statistics were also used to identify and comment on trends and observations during the study period. Clinical follow-ups following digital replantation were not at standardised intervals (i.e. not always exactly at four weeks postoperatively), but all patients were seen by a senior surgeon for a clinical review at four weeks postoperatively or shortly thereafter. According to local guidelines, formal ethics approval was not required for this retrospective analysis. All patient data was stored on a password-protected document and has been anonymised to maintain confidentiality.

## Results

A total of 21 patients with 39 traumatic digital amputations were included for analysis. The mean age of patients was 38.6 years (SD 18.34; range 3 to 68 years). Seven digits were deemed unsalvageable and were immediately terminalised, resulting in 32 replantation attempts in 21 patients. The injured digit varied between cases and is summarised in Table 1. The level of amputation for injured digits is summarised in Table 2. Twenty-three replantation attempts (71.88%; n=23/32) were made within normal working hours. Of those, nine (28.13%; n=9/32) were on the same day, and 14 (43.75%; n=14/32) were delayed until the next day. Nine replants were attempted outside of normal working hours (28.13%; n=9/32). 

**Table 1:** Digit in which replantation was attempted.

Digit Injured	Frequency
Thumb	10
Index	7
Middle	6
Ring	6
Little	3

**Table 2:** Level of injury of digit in which replantation attempted.

Level of injury	Number of digits
Distal interphalangeal joint (DIPJ)	2
Middle phalanx (P2)	3
Proximal interphalangeal joint (PIPJ)	10
Proximal phalanx (P1)	6
Metacarpophalangeal joint (MCPJ)	6
Proximal to MCPJ	5

Success rate of replantation 

The overall success rate across all replantation attempts was 68.75%(n=22). Digital replantation performed during working hours (either the same day or the next day from injury) was more successful (66.67%; n=9 and 78.57%; n=14, respectively). Replantation attempts made out of hours were found to be less successful (55.55%; n=9). Statistical analysis was performed to compare success rates between groups. No statistically significant difference in success was appreciated between groups (all p values > 0.05). A summary of results can be found in Table 3 and results of statistical analysis can be found in Table 4.

**Table 3:** Success rate, rate of revision and success rate of revision surgery in hours vs. out of hours.

Time of Replantation Attempt	Number of Digit Replants Attempted	Number of Successful Digital Replants	Success Rate of Replantation Attempts (%)	Number of Replant Attempts Requiring Revision (%)	Success Rate Following Revision (%)
In working hours	23	17	73.91%	26.09%	50%
Same day in working hours	9	6	66.67%	22.22%	50%
Next day in working hours	14	11	78.57%	28.57%	50%
Out of working hours	9	5	55.56%	33.33%	66.67%
Overall (anytime)	32	22	68.75%	28.13%	55.56%

**Table 4:** Comparison of success rate between timing groups using Fisher's exact test

Timing of replantation attempt	Fisher's exact test p-value for comparison of success rate
Same day in working hours vs out of working hours	1
Next day in working hours vs out of working hours	0.36
Same day in working hours vs next day in working hours	0.64

Rate of revision surgery 

Overall, a total of nine digits required one or more revisions (28.13% of all replantation attempts). The revision rate was lowest for those operated on the same day within working hours (22.22%; n=2). The revision rate for those operated the next day within working hours was 28.57% (n=4), and for those operated out of hours, a revision rate of 33.33% (n=3) was observed. Overall, 55.56% (n=5) of replantation attempts requiring revision were successful. A summary of results for revision surgery can be seen in Table 3. 

Length of operation 

Predictably, the average time elapsed between presentation and theatre was shortest for those operated same day (4.31 hours; SD 1.30) and longest for those where surgery was delayed until the next working day (15.19 hours; SD 2.51). Overall, the average length of the operation was 8.13 hours (SD 4.16). The overall operating time per injured digit was 5.93 hours (SD 2.79). Operating time per digit was shortest for those operated within working hours, either the same day or the next day (5.52 hours; SD 1.85 and 4.81 hours; SD 2.52, respectively). The average operating time per digit out of hours was 7.11 hours (SD 3.12). A summary of results for time to theatre and length of operation can be seen in Table 5. 

**Table 5:** Time to theatre and length of operation

Time of Replantation Attempt	Mean Time to Theatre (hours) (SD)	Mean Operation Time (hours) (SD)	Mean Operating Time per Digit (hours) (SD)
In working hours (Same or next day)	11.56 (SD 5.76)	8.9 (SD 4.78)	5.05 (SD 2.26)
Out of working hours	7.03 (SD 2.26)	7.11 (SD 3.12)	7.11 (SD 3.12)
Same day in working hours	4.31 (SD 1.30)	11.88 (SD 2.60)	5.52 (SD 1.85)
Next day in working hours	15.19 (SD 2.51)	7.41 (SD 5.04)	4.81 (SD 2.52)
Overall (anytime)	9.62 (SD 5.06)	8.13 (SD 4.16)	5.93 (SD 2.79)

Use of vein grafts 

Vein grafting was used in a total of nine cases (42.85%). Vein grafts were used in 75% (n=3) of cases taking place same-day in-hours, 22.22% (n=2) of cases taking place out-of-hours, and 50% (n=4) of cases taking place next-day in-hours. 

Mechanism of injury 

The most common mechanism of injury was a saw injury (n=16; which included circular saw, band saw, and table saw), followed by woodchipper/splitter injuries (n=3 and 4, respectively). The lowest success rates were observed in meat grinder and crush injuries (0%), but these were represented by only one digit each. Of the 16 saw injuries, 75.56% had a successful digital replantation. A summary of the mechanism of injury and success rate can be seen in Table 6. 

**Table 6:** Summary of the mechanism of injury for digits in which replantation was attempted and success rate of replantation.

Mechanism of Injury for Replant Attempts	Number of Digits on Which Replant Attempted	Number of Successful Replants	Success Rate of Replantation Attempt (%)
Wood splitter	3	2	67%
Woodchipper	4	4	100%
Circular saw	10	6	60%
Band saw	3	3	100%
Table saw	3	2	67%
Crush injury	1	0	0%
Gardening secateurs	2	1	50%
Motorbike/bicycle chain	3	2	67%
Rope avulsion injury	1	1	100%
Sheet metal	1	1	100%
Meat grinder	1	0	0%

## Discussion

The BSSH GIRFT guideline is largely based on the evidence presented by Cavadas et al. who demonstrated that delaying replantation until the following morning did not significantly affect success rates [3]. Breahna et al. also demonstrate that daytime replantation, performed by rested and routine staff, was associated with improved outcomes [4]. Harbour et al. challenge the notion that ischaemia time is such a critical factor influencing replant success, and suggest that this position is based mostly on anecdotal evidence [5]. Overall success or survival rates for digital replantation in the literature range from 48% to 97% [6-8]. In contrast to the above literature, Lin et al. reported no significant association between the timing of replantation and success, noting that overnight replantation was linked to fewer complications and shorter operating times than those performed during the day [9]. Elsewhere in the literature, there is substantial evidence from other surgical specialties suggesting that out-of-hours procedures are associated with higher complication rates and lower survival [10-12]. For this reason, the 'National Confidential Enquiry into Peri-operative Deaths' (NCEPOD) in the UK recommended limiting non-essential out-of-hours operations. The rationale behind this was to increase senior surgeon involvement in all cases and optimise working conditions to improve patient safety and clinical outcomes [13-15].

In most cases during the study period (71.88%; n=23), clinical practice adhered to the recommendation outlined by BSSH GIRFT regarding the timing of digital replantation. A total of 14 replantation attempts (43.75%) were delayed until the next day rather than being taken immediately to the theatre (out of hours). This reflects the prevailing opinion that it is acceptable, and often preferable, to delay surgery until the next day if it is felt that this would improve the chances of a successful replantation. A total of nine replantation attempts however were made outside of working hours (28.12%). On review of these cases, it was not always documented why the decision was made to proceed out of hours. All cases operated overnight, however, involved only a single digit and it is possible that surgeons are more likely to proceed out of hours if the case is deemed to be of lower complexity or duration. One of the key concerns regarding out-of-hours surgery is the limited availability of suitably skilled and experienced staff during night shifts. These team members may be less familiar with required equipment and locally established best practices, which could negatively impact outcomes. It is well known that fatigue during night shifts can impair both individual and team performance which can potentially contribute to poor outcomes [16]. The decisions made by a surgeon regarding the timing of digital replantation are nuanced, and clearly, there remains some discrepancy in clinical practice based on individual preference. It is likely that a surgeon’s previous experience with digital replantation and their situation on the digital replantation learning curve [17], could have an impact on decision-making. It is important to note that the delay of replantation until the following morning can potentially have an impact on the department's provision of normal clinical activity if clinics or elective surgical lists need to be cancelled.

The overall success rate of digital replantation in our study was 73.91% (n=17) during working hours (same or next day) and 55.56% (n=5) out of working hours. Our experience supports the recommendation that a delay of up to 24 hours does not result in harm or negatively impact the success rate of replantation. Comparison of success rate between timing groups revealed no statistically significant difference. However, the highest success rate was observed in cases attempted during the next working day (within 24 hours), which would support the majority of existing literature [3-5]. Given the complexity and technical challenge of digital replantation and microvascular surgery, it is likely that the surgical environment and human factors can significantly influence outcomes. It was noted that the operating time per digit was longest in the out-of-hours group (mean = 7.11 hours per digit) which may have resulted from surgeons having to perform complex surgery in a suboptimal environment. This finding aligns with the BSSH recommendation that it is preferable to delay replantation in favour of a familiar environment with a well-rested, well-briefed, and well-prepared team. 

Revision surgery is accepted as a reasonable and effective salvage technique for digits that are failing [18,19]. In our study, a standardised postoperative protocol was followed. This included half-hourly finger observations for four hours, then hourly finger observations for 24 hours, then two hourly thereafter. If any concerns were raised, this prompted assessment by a senior surgeon and a decision was made regarding whether return to theatre was needed for revision surgery. Analysis of our data revealed that the lowest rate of revision was observed in cases performed on the same day during working hours (22.22%; n=2). This can be compared with the revision rates for those operated on the next day and those operated on the same day out of hours (28.57%; n=4 and 33.33%; n=3 respectively). The revision surgery rate was reasonably comparable between groups but notably higher in those who were operated out of hours. A total of 55.56% (n=5/9) of replantation attempts requiring revision were successful. 

Interposition vein grafting was used in a total of nine cases (42.86%). The influence of vein graft use on ultimate success rate of digital replantation was not examined in this study but both Yan et al. and Smith et al. have previously demonstrated no significant difference in survival rates between digital replantation with or without vein grafting [20,21]. It is well known that vein grafting can significantly extend operating time. In this study, vein grafts were used more frequently during working hours (75%; n=3) than out of hours (22.22%; n=2). This difference could be attributed to surgeons feeling more inclined and supported to use vein grafts when working with a dedicated, consistent and well-trained team, with all necessary equipment, during normal working hours. In contrast, an on-call surgeon operating overnight is likely to be fatigued and keen to minimise operation time and complexity when facing the challenges of out-of-hours surgery. 

The most common mechanism of digital amputation observed was saw blade injuries. The lowest success rates of replantation were observed in meat grinder and crush injuries (0%), but these injuries were represented by only one digit each. The overall success rate for saw injuries was 68.75% (n=11/16). This finding aligns with the experience of Sebastin et al., who demonstrated that clean-cut injuries have a significantly higher success rate compared to crush injuries [22].

This study has several limitations. The data was collected from a single UK centre over a two-year period. Despite the centre being a regional hand trauma facility, the number of digital replantation cases was relatively small (n=32 in 21 patients), which limited the statistical power of our analyses and generalisability of our findings. The timing classification for replantation attempts was based on the start time of surgery, yet with an average operation time of 5.93 hours per digit, surgeries often spanned both working and non-working hours. The retrospective nature of the study introduced the potential for selection bias but our study design aimed to minimise this. Future research on this subject should ideally capture larger samples and if possible utilise prospective methodology. The incorporation of larger sample sizes will be essential to identify subtle differences in outcomes between groups, and draw more definitive conclusions regarding the timing of digital replant attempts and its impact on success. 

## Conclusions

An analysis of two years of clinical practice suggests that our center largely adhered to the GIRFT BSSH guidelines regarding the timing of digital replantation, with some exceptions where surgery was undertaken outside of regular working hours. Digital replantation performed during working hours was associated with shorter operating times per digit and higher overall success rates; however, these differences did not reach statistical significance. Interposition vein grafts were used less frequently outside normal working hours, and potential reasons for this are discussed. Our findings align with the BSSH GIRFT recommendation that digital replantation can reasonably be postponed to the next day (within 24 hours) without a statistically significant reduction in success rate. Further studies using prospective methodologies and larger sample sizes are needed to draw more robust conclusions on this subject.

## References

[1] Crowe CS, Massenburg BB, Morrison SD (2020). Global trends of hand and wrist trauma: a systematic analysis of fracture and digit amputation using the Global Burden of Disease 2017 Study. Inj Prev.

[2] British Society for Surgery of the Hand GIRFT guideline (2001). GIRFT: Traumatic amputations of the digits amputations. https://www.bssh.ac.uk/_userfiles/pages/files/professionals/girft/girft-amputations.pdf.

[3] Cavadas PC, Rubí C, Thione A, Pérez-Espadero A (2018). Immediate versus overnight-delayed digital replantation: comparative retrospective cohort study of survival outcomes. J Hand Surg Am.

[4] Breahna A, Siddiqui A, Fitzgerald O'Connor E, Iwuagwu FC (2016). Replantation of digits: a review of predictive factors for survival. J Hand Surg Eur Vol.

[5] Harbour PW, Malphrus E, Zimmerman RM, Giladi AM (2021). Delayed digit replantation: what is the evidence?. J Hand Surg Am.

[6] Yu H, Wei L, Liang B, Hou S, Wang J, Yang Y (2015). Nonsurgical factors of digital replantation and survival rate: a metaanalysis. Indian J Orthop.

[7] Shaterian A, Rajaii R, Kanack M, Evans GR, Leis A (2018). Predictors of digit survival following replantation: quantitative review and meta-analysis. J Hand Microsurg.

[8] Chang MK, Lim JX, Sebastin SJ (2024). Current trends in digital replantation-a narrative review. Ann Transl Med.

[9] Lin IF, Yoon AP, Kong L, Wang L, Chung KC (2022). Association between daytime vs overnight digit replantation and surgical outcomes. JAMA Netw Open.

[10] van Zaane B, van Klei WA, Buhre WF (2015). Nonelective surgery at night and in-hospital mortality: prospective observational data from the European Surgical Outcomes Study. Eur J Anaesthesiol.

[11] Meewisse AJ, Gribnau A, Thiessen SE, Stenvers DJ, Hermanides J, van Zuylen ML (2024). Effect of time of day on outcomes in elective surgery: a systematic review. Anaesthesia.

[12] Halvachizadeh S, Teuber H, Cinelli P, Allemann F, Pape HC, Neuhaus V (2019). Does the time of day in orthopedic trauma surgery affect mortality and complication rates?. Patient Saf Surg.

[13] (2001). Report of the National Confidential Enquiry into Patient Outcome and Death. https://www.ncepod.org.uk/1995_6.html.

[14] (2001). Who operates when? II. https://www.ncepod.org.uk/2003wow.html.

[15] Faiz O, Banerjee S, Tekkis P, Papagrigoriadis S, Rennie J, Leather A (2007). We still need to operate at night!. World J Emerg Surg.

[16] Cortegiani A, Ippolito M, Misseri G (2020). Association between night/after-hours surgery and mortality: a systematic review and meta-analysis. Br J Anaesth.

[17] Hustedt JW, Nystrom NA, Champagne L (2024). The learning curve in digital replant surgery: 46 prospectively collected cases from a single surgeon over a 10-year period. Cureus.

[18] Hatchell AC, Sandre AR, McRae M, Farrokhyar F, Avram R (2019). The success of salvage procedures for failing digital replants: a retrospective cohort study. Microsurgery.

[19] Güntürk ÖB, Kayalar M, Bali U, Özaksar K, Toros T, Gürbüz Y (2020). Clinical outcomes of salvage revision surgery following finger replantation with vascular insufficiency: a retrospective study. Acta Orthop Traumatol Turc.

[20] Yan H, Jackson WD, Songcharoen S (2009). Vein grafting in fingertip replantations. Microsurgery.

[21] Smith AC, Nikkhah D, Wade R (2021). Survival statistics of digital replantation in the UK. Cureus.

[22] Sebastin SJ, Chung KC (2011). A systematic review of the outcomes of replantation of distal digital amputation. Plast Reconstr Surg.

